# An internal ribosome entry site element directs the synthesis of the 80 kDa isoforms of protein 4.1R

**DOI:** 10.1186/1741-7007-6-51

**Published:** 2008-12-04

**Authors:** Eva Lospitao, Carmen M Pérez-Ferreiro, Altea Gosálbez, Miguel A Alonso, Isabel Correas

**Affiliations:** 1 Departamento de Biología Molecular, Universidad Autónoma de Madrid y Centro de Biología Molecular Severo Ochoa, Consejo Superior de Investigaciones Científicas, Nicolás Cabrera, 1. Cantoblanco. E-28049 Madrid, Spain

## Abstract

**Background:**

In red blood cells, protein 4.1 (4.1R) is an 80 kDa protein that stabilizes the spectrin-actin network and anchors it to the plasma membrane through its FERM domain. While the expression pattern of 4.1R in mature red cells is relatively simple, a rather complex array of 4.1R protein isoforms varying in N-terminal extensions, internal sequences and subcellular locations has been identified in nucleated cells. Among these, 135 kDa and 80 kDa isoforms have different N-terminal extensions and are expressed either from AUG1- or AUG2-containing mRNAs, respectively. These two types of mRNAs, varying solely by presence/absence of 17 nucleotides (nt) which contain the AUG1 codon, are produced by alternative splicing of the 4.1R pre-mRNA. It is unknown whether the 699 nt region comprised between AUG1 and AUG2, kept as a 5' untranslated region in AUG2-containing mRNAs, plays a role on 4.1R mRNA translation.

**Results:**

By analyzing the *in vitro *expression of a panel of naturally occurring 4.1R cDNAs, we observed that all AUG1/AUG2-containing cDNAs gave rise to both long, 135 kDa, and short, 80 kDa, 4.1R isoforms. More importantly, similar results were also observed in cells transfected with this set of 4.1R cDNAs. Mutational studies indicated that the short isoforms were not proteolytic products of the long isoforms but products synthesized from AUG2. The presence of a cryptic promoter in the 4.1R cDNA sequence was also discounted. When a 583 nt sequence comprised between AUG1 and AUG2 was introduced into bicistronic vectors it directed protein expression from the second cistron. This was also the case when ribosome scanning was abolished by introduction of a stable hairpin at the 5' region of the first cistron. Deletion analysis of the 583 nt sequence indicated that nucleotides 170 to 368 are essential for expression of the second cistron. The polypyrimidine tract-binding protein bound to the 583 nt active sequence but not to an inactive 3'-fragment of 149 nucleotides.

**Conclusion:**

Our study is the first demonstration of an internal ribosome entry site as a mechanism ensuring the production of 80 kDa isoforms of protein 4.1R. This mechanism might also account for the generation of 60 kDa isoforms of 4.1R from a downstream AUG3. Our results reveal an additional level of control to 4.1R gene expression pathways and will contribute to the understanding of the biology of proteins 4.1R and their homologues, comprising an ample family of proteins involved in cytoskeletal organization.

## Background

The mammalian proteome has been estimated to be at least an order of magnitude larger than its gene number. This highlights the importance of determining which genes give rise to protein diversity and the mechanisms involved in the generation of protein diversity from single genes.

The protein 4.1R gene, *EPB41*, is an example of a gene generating protein diversity. It is best known for encoding protein 4.1R, originally identified as an 80 kDa component of the membrane skeleton of human red blood cells. In these cells, protein 4.1R stabilizes the spectrin-actin network and mediates its attachment to the overlying lipid bilayer through interactions with integral membrane proteins [1]. While the expression pattern of 4.1R in mature red cells is relatively simple, a rather complex array of 4.1R protein isoforms of varying sizes [2,3] and different subcellular locations [4-6] has been reported in nucleated cells, indicating that protein 4.1R plays roles at multiple sites in the cell. The roles and partners of 4.1R in non-erythroid cells are beginning to be elucidated. It turns out that they play structural roles, organizing membrane protein domains and/or linking membranes to internal cytoskeletal and nucleoskeletal networks [6-15]. Protein 4.1R is the founding member of a large family of proteins, the band 4.1 superfamily, containing a highly conserved region designated 'the FERM domain'. The domain takes its name from the 4.1 (four point one) and ERM (ezrin, radixin moesin) proteins where it was discovered. Protein 4.1R and three homologues of 4.1R, namely 4.1B (abundant in brain), 4.1G (general distribution) and 4.1N (abundant in neurons), constitute the protein 4.1 family. FERM-containing proteins comprise a diverse group of eukaryotic proteins that bind membrane proteins and lipids and some of the members (for instance, ERMs, talin, focal adhesion kinase, proteins 4.1) are also involved in the organization of the actin cytoskeleton [1].

The complex *EPB41 *gene is approximately 240 kb long and is subject to extensive regulation at the level of alternative pre-mRNA splicing [16-18]. The regulated combinatorial use of at least 10 internal coding exons of the 4.1R gene is responsible for the extensive range of 4.1R isoforms, which differ with respect to their internal amino acid sequences. Additionally, two types of 4.1R isoforms varying in their N-terminal extensions can be generated if exon 2' is maintained or spliced out. It is well known that inclusion of exon 2' retains the upstream AUG (AUG1) translation-initiation codon responsible for the synthesis of the long isoforms of 4.1R protein, (~135 kDa or 4.1R^135^). Short isoforms (~80 kDa or 4.1R^80^) are generated from mRNAs from which exon 2' has been spliced out and translation is initiated at a downstream start site (AUG2) present in exon 4 [19-21]. Recent studies have revealed great complexity in the 5' region of the 4.1R gene and have shown evidence of coupling between transcription and alternative splicing that directly affects alternative splicing of exon 2', thus regulating the synthesis of structurally different 4.1R isoforms in various cell types [22,23]. A major challenge in the field is to explore the mechanisms by which a single 4.1R gene can express such a variety of isoforms with diverse structures and functions.

Regulation of translation plays a key role in gene expression control. Translation of eukaryotic mRNAs is mainly initiated by a linear scanning mechanism [24]. Several less commonly used alternatives have also been identified that permit, in some cases, the production of isoforms differing in their N-terminal extensions [25-27]. Indeed, alternative translation initiation at different in-frame start codons is a process by which a single mRNA gives rise to proteins varying in their amino terminal extensions. Specific mRNAs bypass the linear ribosome scanning process by directly recruiting the ribosome and positioning it at the start codon through internal ribosome entry site (IRES) sequences. IRES elements were first discovered in 1988 in RNA of poliovirus and encephalomyocarditis virus [28,29], two members of the *Picornaviridae *family. More recently, IRES activities have been detected in an increasing number of cellular mRNAs from yeast, *Drosophila*, birds and mammals, showing that the IRES process is far more extensive than previously thought. IRES-containing mRNAs encode a variety of proteins such as translation initiation factors, transcription factors, oncogenes, growth factors, homeotic genes and survival proteins [30]. IRES and IRES trans-acting factors (ITAFs) collaborate in the recruitment of the 40S ribosomal subunit. It has been proposed that there are both specific ITAFs, which control the activity of related groups of IRESs, and general ITAFs (for example, polypyrimidine tract-binding protein, PTB) [31].

In this study we show that 4.1R mRNAs containing the AUG1 responsible for the synthesis of the long isoforms (~135 kDa) have an IRES element within the sequence located between the AUG1 and the AUG2 that allows the use of this internal site and hence the synthesis of the short (~80 kDa) 4.1R isoforms. The well-known ITAF, PTB, binds to the IRES-containing sequence. Our data reveal that: (i) AUG1/AUG2-containing mRNAs can give rise to both long and short isoforms of protein 4.1R; (ii) the mechanism involved in the synthesis of the short isoforms is an internal entry of the ribosome; (iii) the short isoforms of the 4.1R protein can be generated from two different transcripts, those containing the AUG1 (shown here) and those lacking the AUG1 but containing the AUG2 [19-21]. These data suggest that the short (~80 kDa) isoforms of protein 4.1R are essential to the cell and that diverse mechanisms have therefore evolved to ensure their existence.

## Results

### Two proteins are synthesized *in vitro *from a specific set of 4.1R cDNAs

**Figure 1:**
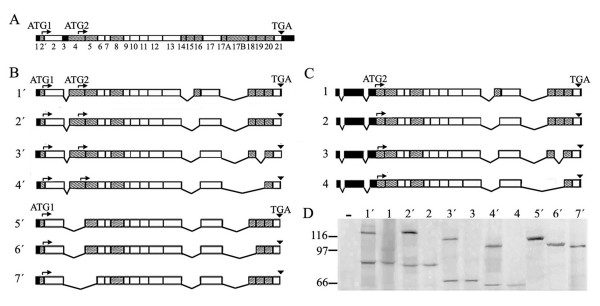
**Analysis of the protein products synthesized *in vitro *from 4.1R cDNAs**. A. Schematic representation of the exon map for the 4.1R protein. Exons are coded as follows: *striped*, alternative; *white*, constitutive; *black*, non-coding. The number of each exon is shown at the bottom. Two translation-initiation sites at exons 2' (ATG1) and exon 4 (ATG2) are indicated, as is the stop codon (TGA) at exon 21. B and C, exon map of the 4.1R^135 ^cDNAs (B) and the 4.1R^80 ^cDNAs (C) used in the *in vitro*-coupled transcription and translation assays. D. *In vitro*-coupled transcription and translation reactions of the indicated 4.1R cDNAs. Reaction products were labelled with [^35^S] methionine and autoradiographed. A control reaction containing all components of the mixture except the cDNA template is also shown (-).

In this study we analyzed the *in vitro *expression of eleven 4.1R cDNAs, previously cloned by our group [32,33], by coupled *in vitro *transcription and translation reactions using a T7 reticulocyte lysate system. Of the eleven 4.1R cDNAs, seven contained exon 2' (4.1R^135 ^cDNAs) and, therefore, the upstream translation initiation site ATG1, which is used for the synthesis of long isoforms of protein 4.1R (Figure 1A and Figure 1B). The other four cDNAs lacked exon 2' (4.1R^80 ^cDNAs) and hence give rise to short isoforms of protein 4.1R by using the downstream ATG2 present in exon 4 as a translation initiation site (Figure 1A and Figure 1C). For simplicity, we will name 1' to 7' the set of exon 2'-containing cDNAs and 1 to 4 the set lacking exon 2'. It should be mentioned that the long isoforms of protein 4.1R are generally referred to as 4.1R^135 ^or ~135 kDa, although their sizes range from ~97 kDa to 135 kDa. Similarly, the short isoforms of protein 4.1R are generally referred to as 4.1R^80 ^or ~80 kDa, although their sizes range from ~62 kDa to 80 kDa.

Focusing on the results of the *in vitro *expression of cDNAs 1' to 7', we observed that all of them gave rise to the expected ATG1-translated proteins (Figure 1D) and, in addition, four of them produced a second product of faster electrophoretic mobility (Figure 1D, lanes 1', 2', 3' and 4'). By analyzing the exon composition of cDNAs 1' to 7', we noticed that those giving rise to two products all included exon 4 (Figure 1B). Notably, cDNAs 2' and 5' generated two and one 4.1R product, respectively, and differed only by the presence (cDNA 2') or absence (cDNA 5') of exon 4 (Figure 1B). cDNAs 1 to 4 use the ATG2 present in exon 4 as a translation initiation site and their exon composition is similar to that of cDNAs 1' to 4', apart from lacking exon 2', so we compared the size of their *in vitro*-expressed products. Figure 1D shows that the size of the ATG2-translated products originating from cDNAs 1 to 4 was identical to that of the faster-migrating product synthesized from cDNAs 1' to 4'. These results suggest that the ATG2 site is probably used in 4.1R^135 ^cDNAs to synthesize the short 4.1R products.

### The short protein synthesized *in vitro *from 4.1R^135 ^cDNAs is generated from the ATG2 site

**Figure 2:**
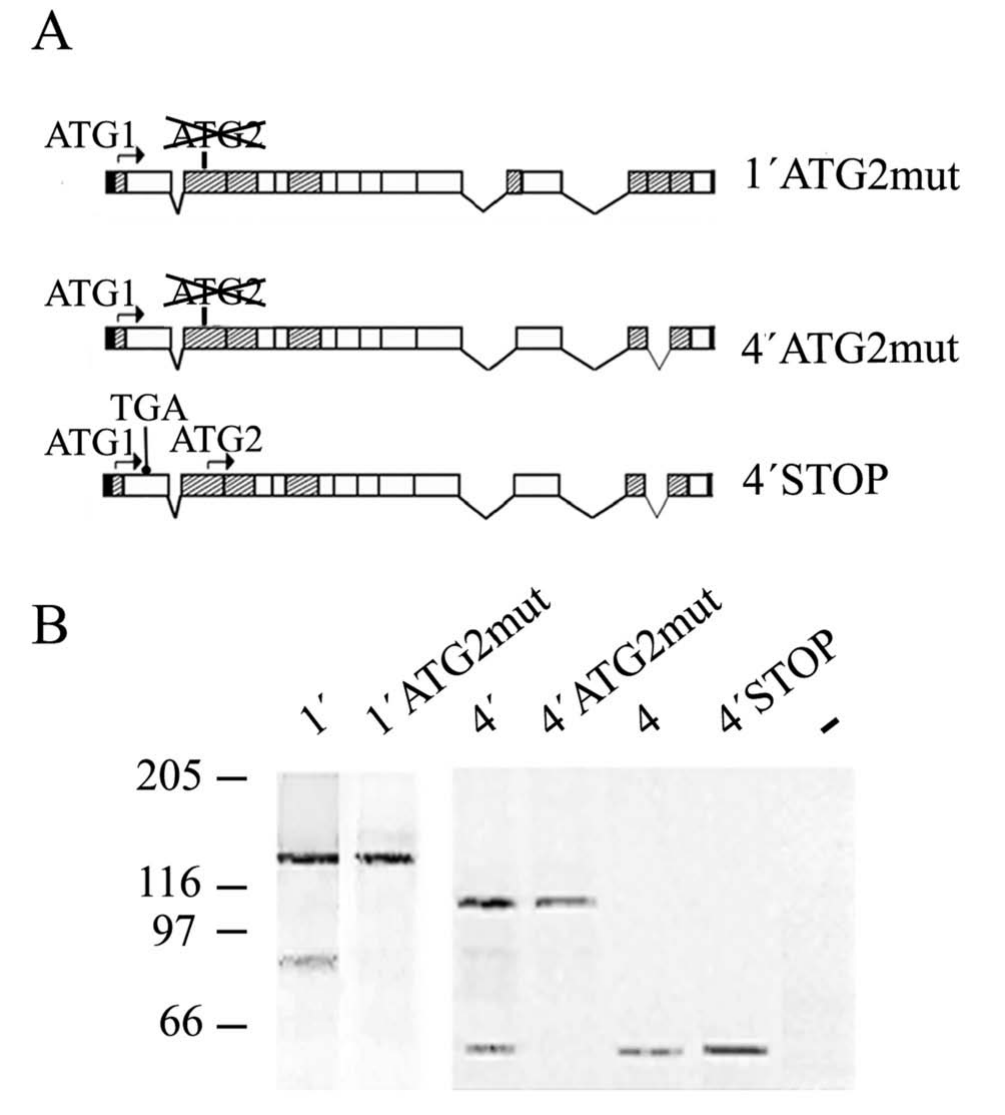
**The short protein product is synthesized from the ATG2 present in exon 4 and is not a proteolytic fragment from the long protein product**. A. Schematic representation of the 4.1R^135 ^mutant cDNAs used in the *in vitro *assays shown in B. The ATG2 was replaced by GTG in cDNAs 1' and 4' thus generating 1'ATG2mut and 4'ATG2mut, respectively. In the third mutant, a TGA stop codon was introduced between exons 2 and 4 of cDNA 4' (4'STOP). B. Autoradiograph showing the products obtained from the *in vitro*-coupled transcription and translation reactions of the indicated 4.1R cDNAs. Lanes 1', 4'and 4 correspond to the *in vitro *products from the wild type cDNAs 1', 4'and 4, respectively.

We next performed a directed mutagenesis experiment to replace the ATG2 codon by GTG in two of the 4.1R^135 ^cDNAs, 1' and 4' (Figure 2A, 1'ATG2mut and 4'ATG2mut). The mutant cDNAs were *in vitro*-transcribed and translated, and the synthesized products analyzed by autoradiography (Figure 2B). The mutation completely abolished the synthesis of the short 4.1R product from both 4.1R^135 ^cDNAs, whereas the synthesis of the ATG1-translated product remained unchanged (compare lanes 1' and 1'ATG2mut and lanes 4'and 4'ATG2mut). These results are consistent with the hypothesis that the short protein originates from 4.1R^135 ^cDNAs translated from the downstream ATG2 site.

### The short protein synthesized *in vitro *from 4.1R^135 ^cDNAs is not produced by proteolysis

The results described above also suggest that the short 4.1R product is unlikely to be produced from proteolytic cleavage of the long 4.1R protein. However, to rule out this possibility definitively we introduced a stop codon in cDNA 4' upstream of the ATG2 by site-directed mutagenesis (Figure 2A, 4'STOP). Figure 2B shows that, indeed, in the absence of the full-length ATG1-translated product, the short protein product was still generated (4'STOP). Additionally, when the 4.1R^135 ^cDNAs were expressed *in vitro *in the absence of the T7 promoter, no protein products were observed (data not shown).

These results indicate that the 4.1R sequence does not contain a cryptic promoter directing the synthesis of the short protein product, and that the short product does not result from proteolytic cleavage of the long one.

### *In vivo *expression of the set of 4.1R^135 ^cDNAs containing exon 4

**Figure 3:**
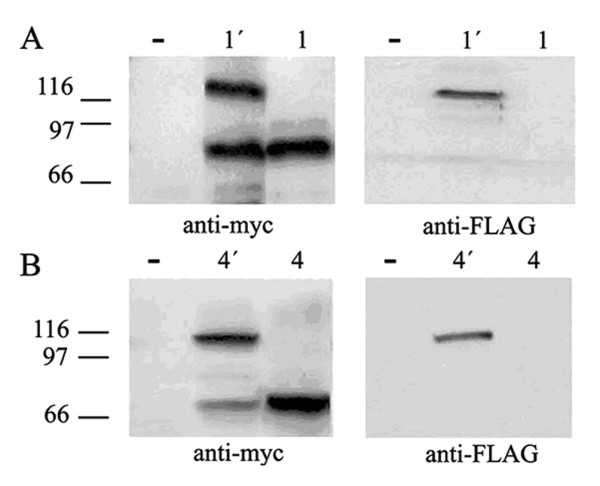
**The short protein 4.1R is also expressed *in vivo *from 4.1R^135 ^cDNAs**. A. Western blot of total protein extracts of COS-7 cells transfected with an empty plasmid (-) or transfected with 4.1R cDNAs 1' and 1 revealed with anti-myc (anti-myc) and anti-FLAG (anti-FLAG) antibodies. B. Western blot of total protein extracts of COS-7 cells transfected with an empty plasmid (-) or transfected with 4.1R cDNAs 4' and 4 revealed with anti-myc and anti-FLAG antibodies. All 4.1R proteins were tagged at the amino and carboxyl termini with FLAG and myc epitopes, respectively, to detect the exogenously expressed proteins 4.1R.

All previous experiments were developed in a coupled *in vitro *transcription-translation reticulocyte lysate system. We next considered whether the ATG2 site of 4.1R^135 ^cDNAs was also used in living cells to give rise to the production of the short 4.1R protein. To investigate this, cDNAs 1', 2', 3' and 4' were independently transfected in COS-7 cells and the expressed proteins were analyzed by western blot. To detect the exogenous ATG1-translated 4.1R protein specifically we used the anti-FLAG antibody, since this epitope was added to the N-terminus of the long 4.1R molecule. To detect both the exogenous ATG1- and ATG2-translated 4.1R proteins we used the anti-myc antibody as this epitope was added to the C-terminus of the 4.1R molecule. Figure 3A and 3B shows representative results obtained for cDNAs 1' and 4' that were similar to those observed for cDNAs 2' and 3' (data not shown). As expected, the long protein was synthesized and recognized by both antibodies (Figure 3A lanes 1' and Figure 3B lanes 4'). The short protein was also synthesized and detected by the anti-myc antibody but not by the anti-FLAG antibody (Figure 3A lanes 1' and Figure 3B lanes 4'). The size of the short protein generated from cDNAs 1' and 4' was similar to that of the ATG2-translated 4.1R isoform synthesized from the corresponding 4.1R^80 ^cDNA (compare Figure 3A lanes 1' and 1 and Figure 3B lanes 4' and 4). These results indicate that the short protein most probably corresponds to the 4.1R product synthesized from the ATG2 site.

Consistent with the results observed *in vitro*, all these data suggest that the ATG2 site present in the set of 4.1R^135 ^cDNAs containing exon 4 is indeed used in living cells.

### Generation of the short 4.1R isoform is not due to a cryptic promoter

**Figure 4:**
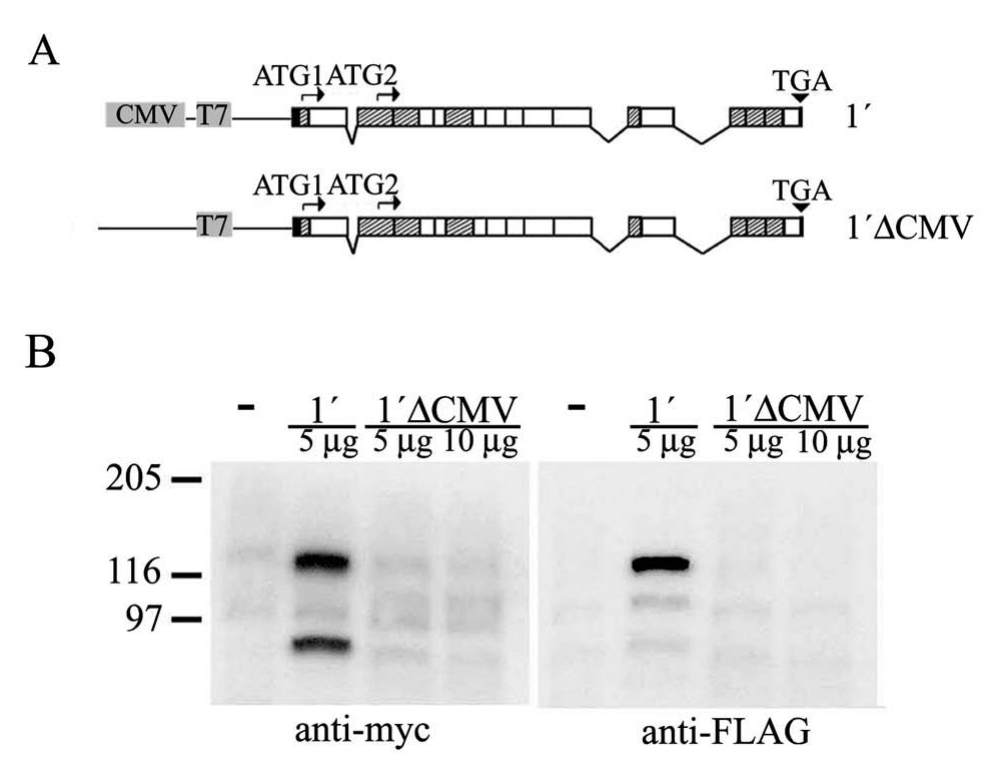
**The generation of the short protein 4.1R is not due to a cryptic promoter**. A. Schematic representation of the plasmids used in the western blot analysis shown in B. The first plasmid contained the 4.1R cDNA 1' under the control of the CMV promoter, whereas the CMV promoter was eliminated from the second plasmid (1'ΔCMV). 4.1R proteins were tagged at the amino and carboxyl termini with FLAG and myc epitopes, respectively, to detect the exogenously expressed 4.1R proteins. B. Western blot of total protein extracts from COS-7 cells transfected with an empty plasmid (-) and from cells transfected with the indicated plasmids, revealed with the anti-myc and the anti-FLAG antibodies.

A cryptic promoter could be responsible for the production of mRNA species giving rise to the short 4.1R protein detected in the immunofluorescence and western blot analyses. To test this possibility we eliminated the cytomegalovirus (CMV) promoter from the construct containing cDNA 1' (Figure 4A, 1'ΔCMV) and performed transfection experiments in COS-7 cells to analyze the protein expression pattern by western blot (Figure 4B) using the anti-myc and the anti-FLAG antibodies. The long and short 4.1R protein bands were observed for the control cDNA 1' construct, whereas removal of the CMV promoter resulted in a lack of synthesis of both 4.1R proteins (Figure 2B, lanes 1'ΔCMV). Thus, the short 4.1R protein detected in COS-7 cells transfected with the set of 4.1R^135 ^cDNAs containing exon 4 is not due to the existence of an internal promoter.

### The 5' sequence upstream of the ATG2 site directs internal translation from a bicistronic vector

**Figure 5:**
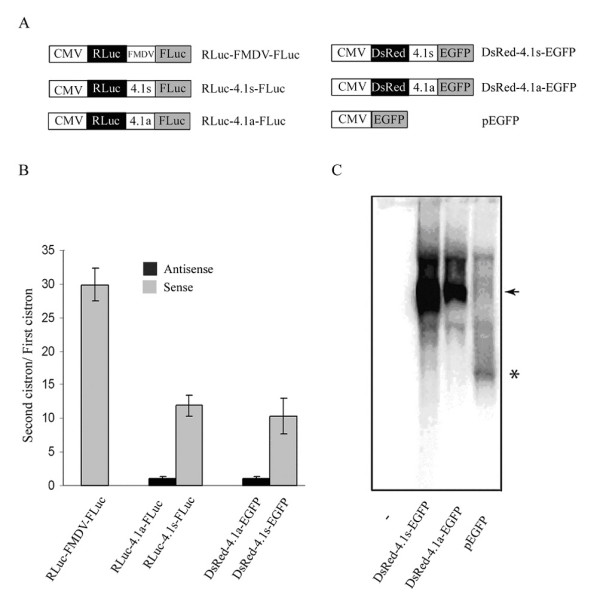
**The 4.1R sequence upstream of the ATG2 directs translation of the second cistron from bicistronic vectors**. A. Schematic representation of the bicistronic and monocistronic constructs used. B. The indicated constructs were transfected into COS-7 cells. Luciferase activity or fluorescence intensity was determined when the bicistronic vectors containing the *Renilla*/firefly luciferase or the DsRed/EGFP reporters, respectively, were used. The firefly:*Renilla *or EGFP:DsRed ratios are expressed relative to that of the plasmids containing the sequence of 4.1R in the antisense orientations (RLuc-4.1a-FLuc and DsRed-4.1a-EGFP, respectively), which were assigned a value of 1. Error bars correspond to the SEM from five independent experiments. C. Total RNA was isolated from COS-7 cells transfected with an empty plasmid (-) or transfected with DsRed-4.1s-EGFP, DsRed-4.1a-EGFP and pEGFP and analyzed by Northern blot using full-length EGFP cDNA as a hybridization probe. The arrowhead indicates the position of the full-length bicistronic RNA and the asterisk that of the EGFP monocistronic RNA.

Having ruled out the existence of a cryptic promoter and proteolytic cleavage as the mechanisms for generating the short 4.1R isoforms, we considered internal translation to be the most plausible mechanism responsible for their synthesis. To test this hypothesis, a sequence comprised of 583 nucleotides upstream of the ATG2 (from nucleotide 39 to 621 in the reference sequence AF156225) of 4.1R was inserted into a bicistronic plasmid between the *Renilla *and firefly luciferase reporter genes (RLuc-4.1s-FLuc). Bicistronic plasmids containing the 583 nucleotides in an antisense orientation (RLuc-4.1a-FLuc) or the foot-and-mouth disease virus (FMDV) IRES (RLuc-FMDV-FLuc) inserted between the two cistrons were used as negative and positive controls, respectively (Figure 5A). These plasmids were independently transfected into COS-7 cells and the expressed proteins were detected by enzymatic activity assays. As shown in Figure 5B, cell lysates transfected with RLuc-4.1s-FLuc were positive for firefly luciferase suggesting that the cloned 583 nucleotides of 4.1R contain a functional IRES. Expression of the second cistron directed by the 4.1R sequence was lower than that directed by the FMDV IRES. A second bicistronic system consisting of DsRed and enhanced green fluorescence protein (EGFP) as first and second reporter cistrons, respectively, corroborated the results obtained with the *Renilla*/firefly luciferase system (Figure 5B). As the fluorescence intensity of DsRed and EGFP is very easily measured by flow cytometry, we adopted this system for the rest of the experiments in this study.

### The process directing translation of the second cistron in the bicistronic assay is not due to RNA processing or RNA cleavage

To investigate the possibility that RNA cleavage or RNA splicing contributed to the expression of the second reporter gene, we examined the integrity of the bicistronic transcript by northern blot analysis (Figure 5C) following transfection of COS-7 cells with the bicistronic vector DsRed-4.1s-EGFP or with DsRed-4.1a-EGFP or with a plasmid coding only for EGFP (pEGFP vector), which was used as monocistronic transcript control (Figure 5A). A band corresponding to the expected size of 2100 bp for the bicistronic RNAs was detected with an EGFP-specific cDNA probe from total RNA extracts isolated from cells transfected with the bicistronic vectors (Figure 5C). Monocistronic EGFP mRNA (720 bp) species were not detected in any of the bicistronic vectors used (compare lanes DsRed-4.1s-EGFP and DsRed-4.1a-EGFP with pEGFP).

### An IRES element located in the region 5' upstream of the ATG2 directs translation of the second cistron

**Figure 6:**
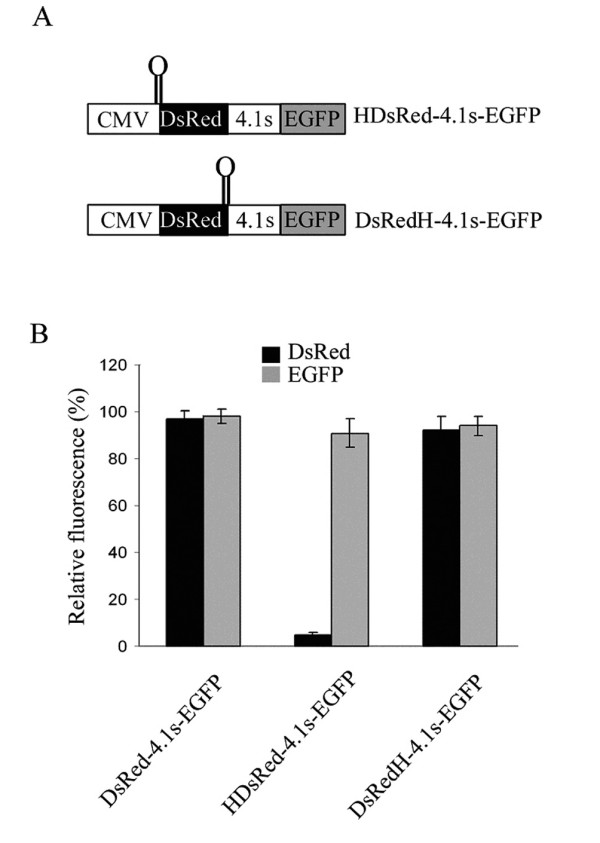
**The 4.1R sequence contains an IRES element driving translation of the second cistron**. A. Schematic representations of the two bicistronic constructs used in which a stable hairpin () that impedes ribosome scanning was cloned upstream (HDsRed-4.1s-EGFP) or downstream (DsRedH-4.1s-EGFP) of the first cistron, DsRed. B. The indicated constructs were transfected into COS-7 cells and their fluorescence intensity determined. Values presented are normalized against the fluorescence intensity produced from the plasmid DsRed-4.1s-EGFP, which was assigned a value of 100. Error bars correspond to the SEM from four experiments.

A positive bicistronic test indicates that an alternate initiation mechanism is involved in the production of the short 4.1R isoform. A number of internal translational initiation mechanisms have been described: reinitiation, leaky scanning, shunting and internal ribosome entry [27]. Reinitiation cannot explain the generation of the short 4.1R isoforms. To distinguish between the other three possibilities, we generated an additional bicistronic construct in which a synthetic stem loop with a ΔG° = -57 kcal/mol, which is known to decrease the scanning of the ribosome [34], was inserted either 5' to the first cistron, HDsRed-4.1s-EGFP, or between the stop codon of the first cistron and the 4.1R sequence, DsRedH-4.1s-EGFP (Figure 6A). These constructs and the original DsRed-4.1s-EGFP construct were independently transfected in COS-7 cells and expression of DsRed and EGFP was determined by flow cytometry. Figure 6B illustrates that the hairpin abolished the expression of the first cistron when added to its 5' end but not the expression of the second cistron (lanes HDsRed-4.1s-EGFP). The second cistron was also expressed when the hairpin was added downstream of the DsRed reporter gene (lanes DsRedH-4.1s-EGFP). These results indicate that the 4.1R sequence comprised between the ATG1 and the ATG2 contains an internal ribosome entry site.

### The length of the 4.1R sequence between the two reporter genes determines the efficiency of the second cistron translation

**Figure 7:**
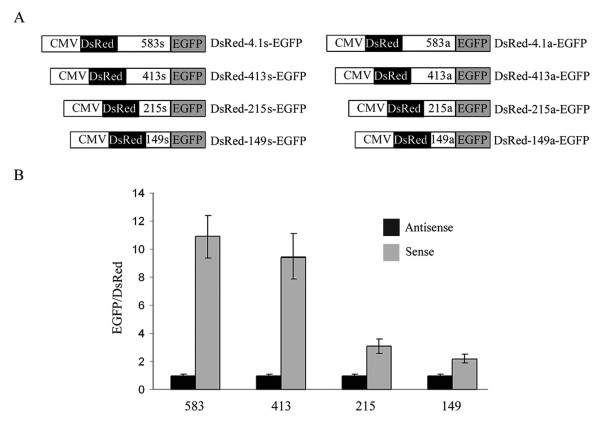
**The length of the 4.1R sequence inserted in the bicistronic vector determines the expression of the second cistron**. A. Schematic representation of the 4.1R sequences introduced into the intercistronic region of the DsRed-EGFP bicistronic vector. Numbers in the intercistronic region (583, 413, 215 and 149) refer to the nucleotides maintained from the 3' region of the 583 nt 4.1R sequence. The fragments were cloned in the sense (s) and antisense (a) orientations. B. The indicated constructs were transfected into COS-7 cells and their fluorescence intensity determined. The EGFP:DsRed ratios are expressed relative to that of the plasmids containing the sequence of 4.1R in the antisense orientations, which were assigned a value of 1. Error bars correspond to the SEM from five experiments.

To determine the influence of the 5' region upstream of the ATG2 on the usage of the second cistron, we prepared three different 5' deletion constructs (Figure 7A, DsRed-413s-EGFP, DsRed-215s-EGFP and DsRed-149s-EGFP). As negative controls, we used the same 4.1R sequences in antisense orientations. All these constructs were transfected in COS-7 cells and fluorescence intensity was analyzed by flow cytometry (Figure 7B). The construct lacking 170 nucleotides from the 5' region of exon 4 (made up of 413 nucleotides) had slightly lower efficiency than the construct with the entire sequence (583 nucleotides). The other two deletion constructs had very low efficiencies, almost comparable with that of their corresponding antisense controls. Thus, removal of 5' sequences negatively affects the translation of the second cistron, being nucleotides 170 to 368 essential for IRES activity.

### The 4.1R sequence containing the IRES interacts with PTB

**Figure 8:**
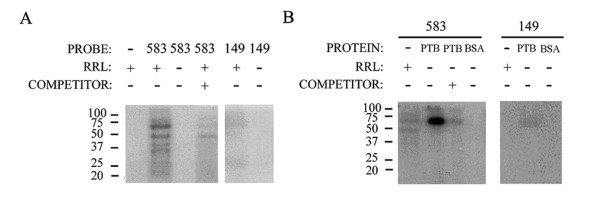
**PTB binds to the 4.1R sequence containing the IRES**. 4.1R radiolabeled transcripts containing (583 nt) or lacking (149 nt) IRES activity (see Figure 7) were used in UV-crosslinking assays with reticulocyte lysate (RRL) proteins (A) or purified recombinant polypyrimidine tract-binding protein (PBT) (B). Following RNase A treatment, proteins were fractionated by 10% SDS-PAGE and autoradiographed. As a control, bovine serum albumin was added at the same molar concentration as PTB. A 150-molar excess of competitor to labeled probe was added where indicated.

Although the precise mechanism by which cellular IRESs promote translation is not fully understood, the participation of IRES-binding proteins, referred to as ITAFs, is well documented [31]. To analyze proteins interacting with the 583 nucleotide segment containing the 4.1R IRES, we performed UV-crosslinking experiments. Reticulocyte lysates were used as a source of ITAFs. As a control we used a 4.1R riboprobe lacking IRES activity, the 149-ribonucleotide fragment corresponding to the 3' terminal end of the 583 ribonucleotide sequence (see Figure 7). Figure 8 shows that the ^32^P-labelled 583 riboprobe crosslinked proteins ranging in size from 22 to 80 kDa which, by contrast, the 149 ribonucleotide fragment did not crosslink. The crosslinking of these proteins was globally competed by incubation with a 150-fold molar excess of the unlabelled 583 riboprobe. A protein of approximately 57 kDa was one of the most prominent bands bound to the 583 riboprobe. As PTB is a ~57 kDa protein that interacts with pyrimidine-rich sequences of many viral and cellular IRESs, we analyzed the possible interaction of PTB with the 583 riboprobe. Figure 8B shows that the 583 but not the 149 riboprobe crosslinked purified, recombinant PTB. This result is consistent with the presence of a polypyrimidine-rich tract in the large fragment, which is absent from the short one.

## Discussion

Non-erythroid cells express a wide range of 4.1R isoforms that vary in N-terminal extensions and in internal sequences. This diversity is mainly generated by mechanisms coupling alternative promoter transcription, alternative splicing of the 4.1R pre-mRNA and the use of different translation-initiation sites [19-22]. We report here an additional layer of complexity in the already complex 4.1R gene regulation pathways based on our finding that alternative internal translation, more specifically an IRES-driven translation, is involved in the generation of the short isoforms of protein 4.1R. Therefore, mammalian 4.1R mRNA is added to the limited number of cellular polycistronic transcripts synthesizing two proteins harbouring different N-terminal regions by initiating translation from two in-frame AUG codons.

In the past, we learnt that two types of 4.1R isoforms with different amino-terminal regions, long and short isoforms, were synthesized from two sets of mRNAs generated by alternative splicing of the 4.1R pre-mRNA [19-21]. The long isoforms are generally referred to as 4.1R^135 ^or 135 kDa although their molecular masses vary from ~97 to 135 kDa. The short isoforms are generally referred to as 4.1R^80 ^or 80 kDa, although their molecular masses vary from ~62 to 80 kDa. The two types of 4.1R mRNAs differ solely by the presence or absence of 17 nt, depending on whether exon 2' was maintained or skipped, respectively, implying that proteins 4.1R^80 ^are synthesized from mRNAs containing a rather long 5' UTR (more than 600 nt). Typically, mRNAs that are translated by the linear ribosome scanning mechanism contain short 5' UTRs; by contrast, mRNAs containing IRES elements keep long 5' UTRs [35]. It has always intrigued us whether the long 5' UTR in the 4.1R mRNAs responsible for the generation of 4.1R^80 ^proteins was maintained because it contained a relevant structured element. The present study demonstrates the existence of an IRES element within this region. The IRES element directs the synthesis of the second cistron in two bicistronic systems (Figure 5) and, more importantly, the synthesis of the short isoforms of proteins 4.1R, both *in vivo *and *in vitro*, (Figures 3 and 1, respectively) from 4.1R mRNAs containing exon 2' and most probably from 4.1R mRNAs skipping exon 2'. A protein that is considered to be a general ITAF, PTB [31], is bound to the IRES-containing region but not to an inactive fragment immediately adjacent to the AUG2 (Figure 8). Our finding that a single 4.1R cDNA can give rise to two 4.1R isoforms, long and short, establishes a new level of complexity that needs to be taken into account in future studies.

Most cellular IRES elements characterized to date are located in 5' UTR and few of them are located in coding regions [36-38]. Interestingly, the IRES element identified in 4.1R is not only derived from within the coding region of the mRNAs containing exon 2' but also in the 5' UTR of 4.1R mRNAs lacking exon 2'.

Some earlier studies analyzing cellular IRES elements did not rule out all the possible mechanisms involved in the generation of a shorter protein and, in some cases, the putative IRES sequence happened to be a cryptic promoter [39]. It should be mentioned that mechanisms responsible for the generation of two isoforms from a single cDNA, such as transcription from a cryptic promoter or proteolytic cleavage from the large protein product of 4.1R, were examined in this study. Our data (Figures 4 and 2, respectively) indicated that the short isoforms of protein 4.1R were not generated by either of these two mechanisms. Similarly, the possible contribution of internal translation mechanisms such as shunting or leaky scanning to the internal initiation of translation was also examined in the bicistronic assay. A hairpin sequence capable of abolishing ribosome scanning was introduced at the 5' region of the first cistron and resulted in the failure to synthesize the first, but not the second, cistron (Figure 6). RNA cleavage and processing were also ruled out (Figure 5C). Our data clearly imply that internal entry of the ribosome to the AUG2 is responsible for the generation of the short isoforms of proteins 4.1R.

Many cellular IRES are relatively inactive in *in vitro *translation systems and, in cell assays, they are activated when cells are submitted to stress conditions or at some stages of the cell cycle. It is of note that large amounts of 4.1R^80 ^isoforms were synthesized from 4.1R^135 ^cDNAs containing exon 4 in *in vitro *translation assays and in *in vivo *transfection experiments performed in non-synchronized cells. Consistently, no altered production of 4.1R^80 ^isoforms was detected when transfected cells were arrested in mitosis with nocodazole and then released by nocodazole wash-out or subjected to etoposide-induced apoptosis, serum starvation or heat-shock treatments (data not shown). These observations imply that the short isoforms of protein 4.1R, 4.1R^80^, may be generated constitutively by internal translation from AUG1/AUG2-containing mRNAs.

Given that the short isoforms of protein 4.1R can be synthesized from their own mRNAs, those lacking exon 2' (AUG1) and containing exon 4 (AUG2), what would then be the significance of their generation also by IRES-mediated translation from mRNAs containing AUG1? It is reasonable to hypothesize that 4.1R^80 ^isoforms must be vital to the cell and that diverse mechanisms have therefore evolved to ensure their existence.

Examples of cellular proteins containing two separate IRES sequences have been described [38]. We [33] and others [40] have shown that a third translation-initiation codon (AUG3) present in exon 8 originates ~60 kDa isoforms of protein 4.1R from 4.1R cDNAs lacking exons 2' (AUG1) and 4 (AUG2). This mRNA species contains a 5' UTR that is too long (more than 900 nt) for a linear ribosome scanning mechanism, which may be compatible with the existence of an IRES element. In some experiments, in addition to the generation of the 135 kDa isoform of protein 4.1R we have detected a 60 kDa band (data not shown) suggesting that, indeed, 4.1R mRNAs may contain more IRES elements than that described in this study.

## Conclusion

The capacity of the 4.1R gene, *EPB41*, to encode and differentially express many 4.1R isoforms by alternatively spliced transcripts differing on translation initiation sites and internal sequences, makes *EPB41 *a paradigm of cellular economy in storing information. Our results show for the first time that, in addition to the regulation at the transcriptional and splicing levels, protein 4.1R generation is also controlled at the translational level. The capacity of a single mRNA species to produce two 4.1R proteins by using an internal ribosome entry site adds a new level of complexity to the already complex regulation of 4.1R expression. Our study emphasizes the need to take into account this new level of regulation to achieve a better understanding of the biology of 4.1R proteins and their homologs, a large group of protein isoforms involved in the organization of the actin and microtubule cytoskeletons.

## Methods

### Cell culture and transfection

COS-7 cells were grown as described [41]. Transfection experiments were performed by electroporation using the Electro Cell Manipulator 600 (BTX, San Diego, CA). Cells were processed 48 hours after transfection.

### Antibodies

Anti-FLAG antibody is a rabbit polyclonal antibody (Sigma, Saint Louis, MO). Anti-c-myc monoclonal antibody 9E10 was obtained from the American Type Culture Collection. Anti-horseradish peroxidase-labelled secondary antibodies were obtained from Southern Biotechnology Associates, Inc. Goat anti-rabbit immunoglobulin G (IgG) (H+L) secondary antibody conjugated to Alexa Fluor 488 was obtained from Molecular Probes.

### 4.1R cDNAs, 4.1R mutant constructs and *in vitro *protein expression

4.1R cDNAs used for *in vitro *and *in vivo *expression were generated by RT-PCR using total RNA from MOLT-4 T cells as described previously [32,33]. These cDNAs encoded 4.1R proteins tagged at the C-terminus with the c-myc epitope. Some of the cDNAs encoded 4.1R proteins also tagged at the N-terminus with the FLAG epitope. The cDNAs were cloned into pCR3.1 (Invitrogen) and verified by sequencing [41].

In two 4.1R cDNAs, previously designated 4.1R^135^Δ16 and 4.1R^135^Δ16,18,19 (for simplicity named in this study cDNAs 2' and 4', respectively) the ATG2 present in exon 4 was mutated and the adenine was substituted by guanosine, thus generating 1' ATG2mut and 4' ATG2mut, respectively. The mutant 4' STOP carried a nucleotide substitution at position 182 of exon 2 that changes the triplet AAG to the stop triplet TAG. These three mutant constructs were obtained using the QuikChange site-directed mutagenesis following the manufacturer's instructions (Stratagene). *In vitro *protein expression was achieved by coupled *in vitro *transcription and translation reactions, as previously described [41].

### Bicistronic vectors

The DsRed coding sequence was amplified by PCR from the pDsRed1-N1 vector (Invitrogen) with the sense primer *Nhe*I-red (5'-CCGGTCGCTAGCATGGTGCGCTCC-3') and the antisense primer *Bgl*II-stop-red (5'-CTAGAGTAGATCTCGCTACAGGAA-3'). The PCR product was cloned between the *Nhe*I and *Bgl*II sites of pEGFP-N1 (Invitrogen).

Different 4.1R sequences, amplified by PCR, were inserted into the *Sac*I site of the bicistronic vector. The sense primers used for the DsRed-4.1s-EGFP, DsRed-413s-EGFP, DsRed-215s-EGFP, DsRed-149s-EGFP construct were 5'-GGCCGGAGCTCCACAGCACCAAC-3', 5'-GACTTGACCGAGCTCAAGGAGCGGACA-3', 5'-CCCAATTGCAGAGCTCGAACCGGAAC-3' and 5'-GCAGAAACAGAGCTCGCTCAGGAAGAAC-3', respectively, and the antisense primer was 5'-GCAGTGCATGAGCTCGTGTTTTCTGATTGG-3' in all cases.

The hairpin structure (CCGGATCGG)_3 _described by Koromilas et al [34] was cloned between the *Xho*I and *Nhe*I sites of DsRed-4.1s-EGFP to generate the DsRed*H*-4.1s-EGFP and the *H*DsRed-4.1s-EGFP constructs, respectively.

The 4.1R sequence amplified with the 5'-GGCCGGAGCTCCACAGCACCAAC-3' and 5'-GCAGTGCATGAGCTCGTGTTTTCTGATTGG-3' primers was cloned into the *Sac*I site of the RLuc-FLuc bicistronic vector in sense (RLuc-4.1s-FLuc) and antisense (RLuc-4.1a-FLuc) orientations.

### Luciferase quantification

Relative IRES activity was quantified *in vivo *as the ratio of the expression of firefly luciferase to that of *Renilla* luciferase in COS-7 cells transfected with the bicistronic constructs. Luciferase expression was determined from 1–2 × 10^5 ^cells using the dual luciferase reporter assay system following the manufacturer's instructions (Promega) in a luminometer (Berthold). Five independent experiments were performed in triplicate.

### Northern blot analysis

Total RNA from COS-7 cells transfected with different constructs was extracted using TRIZOL Reagent (Invitrogen Life Technologies). For northern blot analysis, 20 μg of RNA were denatured in 50% formamide and 2.2 M formaldehyde at 65°C, subjected to electrophoresis in a 1% agarose/formaldehyde gel, and transferred to nylon membranes. RNA samples were hybridized under standard conditions to labelled EGFP cDNA. Final blot washing conditions were 0.5 × SSC/0.1% SDS (1 × SSC = 0.15 M NaCl, 0.015 M sodium citrate, pH 7.0) at 65°C.

### RNA riboprobes

To generate RNA riboprobes, PCR was performed with specific primers for the indicated 4.1R fragments to which additional sequences were added for incorporating the T7 RNA polymerase promoter at the 5' end. Radiolabeled RNA probes were prepared by transcription with T7 RNA polymerase in the presence of 0.08 mM unlabelled rUTP plus 25 μCi of (α-^32^P)UTP (400 Ci/mmol)(Amersham).

### UV cross-linking assays

12.5 μl of rabbit reticulocyte lysates were incubated with radiolabeled probes at 30°C for 30 minutes. The reaction mixtures were exposed to UV (254 nm) (Stratalinker 1800; Stratagene) for 10 minutes on ice. Then 20 units of RNase A was added to the reaction and incubated during 10 minutes at 37°C. For competition experiments, a 150-molar excess of unlabelled RNA was added 10 minutes before the addition of the radiolabeled probe. For PTB-4.1R interaction experiments, 100 ng of recombinant His-PTB (a gift from Dr. J.M. Izquierdo, Centro de Biología Molecular Severo Ochoa, Madrid) was incubated with the appropriate radiolabeled probes. The RNA-protein complexes were resolved by SDS-PAGE.

### Immunofluorescence

COS-7 cells were fixed with 4% formalin (37% formaldehyde solution; Sigma), permeabilized, blocked, incubated with the appropriate antibodies, and processed as described [4]. Controls with primary antibodies omitted were included in each experiment. Preparations were examined under a Zeiss epifluorescence microscope.

### Western blot analysis

Protein samples were separated by SDS-polyacrylamide gel electrophoresis and transferred to Immobilon polyvinylidine difluoride (Millipore) in Tris (tris(hydroxyl-methyl)aminomethane)-borate buffer, pH 8.2. Membranes were processed and developed as described [4].

### Flow cytometry analysis

Transfected cells were detached from the dish and suspended at 0.5–1 × 10^6 ^cells/ml in phosphate-buffered saline, 2 mM EDTA. Samples were analyzed by flow cytometry using an argon laser at 488 and 558 nm to detect EGFP and DsRed expression, respectively, in a Calibur cytometer (Becton-Dickinson). Four to five independent experiments were performed in triplicate.

## Abbreviations

CMV: cytomegalovirus; EGFP: enhanced green fluorescence protein; FERM: four point one, ezrin, radixin and moesin; Fluc: firefly luciferase; FMDV: foot-and-mouth disease virus; IRES: internal ribosome entry site; ITAF: IRES trans-acting factor; PTB: polypyrimidine tract-binding protein; Rluc: *Renilla *luciferase.

## Authors' contributions

EPL carried out experiments shown in Figures 3 to 8. CMP and AG performed experiments shown in Figures 1 and 2. MAA participated in the design of the study and critically read the manuscript. IC conceived and coordinated the study and wrote the manuscript. All authors read and approved the final manuscript.
